# Supplementary material for: Site-selective generation of lanthanoid binding sites on proteins using 4-fluoro-2,6-dicyanopyridine
Source: Magn Reson (Gott). 2022 Sep 13;3(2):169–82. doi: 10.5194/mr-3-169-2022 (PMC10539774; doi:10.5194/mr-3-169-2022)
Supplement: The supplement related to this article is available online at: https://doi.org/10.5194/mr-3-169-2022-supplement. [file mr-3-169-supplement.pdf]

Supplement of Magn. Reson., 3, 169–182, 2022
https://doi.org/10.5194/mr-3-169-2022-supplement
© Author(s) 2022. CC BY 4.0 License.

Supplement of

Site-selective generation of lanthanoid binding sites on proteins using 4-fluoro-2,6-dicyanopyridine

Sreelakshmi Mekkattu Tharayil et al.

Correspondence to: Gottfried Otting (gottfried.otting@anu.edu.au)

The copyright of individual parts of the supplement might differ from the article licence.

Table of contents

Synthesis of β-cysteine

Figure S1. NMR and mass spectra of (*S*)-3-amino-4-mercaptobutanoic acid (β-cysteine)

Synthesis of DCP-(L-Cys)₂

Figure S2. NMR spectra of DCP-(L-Cys)$_2$

Figure S3. Mass spectra of GB1 Q32C before and after the complete assembly of the DCP-(L-Cys)$_2$ tag

Figure S4. Mass spectra of GB1 Q32C before and after tagging reaction

Figure S5. Reactivity of the FDCP tag with cysteine residues of different solvent exposure

Figure S6. Mass spectra showing the reaction of the FDCP tag with cysteine residues followed by subsequent reaction with cysteine

Figure S7. UV/Vis absorption spectrum of DCP-(L-Cys)$_2$

Table S1. PCSs of backbone amide protons generated with $TbCl_3$ and $TmCl_3$ in GB1 Q32C with DCP-Cys_2 tags of different chirality

Table S2. PCSs of backbone amide protons generated with $TbCl_3$ and $TmCl_3$ in GB1 Q32C with DCP tags reacted with penicillamines of different chirality

Table S3. $^1D_{HN}$ RDCs of backbone amides of GB1 Q32C with different DCP-Tm tags

Figure S8. Echo-detected W-band EPR spectra of ERp29 S114C/C157S DCP-(β-Cys)$_2$, MBP T237U/T345U and MBP T237C/T345C

Figure S9. Original DEER traces of Fig. 4 in the main text

Figure S10. Distance distributions of Fig. 4 analysed by AI

Figure S11. Raw DEER traces of Fig. 5 in the main text

Figure S12. Distance distribution of MBP T237C/T345C (Fig. 5 of the main text) analysed by AI

Figure S13. Conformation of the DCP-(β-Cys)$_2$-Gd tag used for modelling distance distributions
Figure S14. Gd^{3+}–Gd^{3+} distance distributions for DCP-(L-Cys)$_2$-Gd tags calculated with the program PyParaTools
Figure S15. Gd^{3+}–Gd^{3+} distance distributions for DCP-(β-Cys)$_2$-Gd tags calculated with the program PyParaTools
Figure S16. Diffusion experiment of DCP-(L-Cys)$_2$ in the presence of YCl_3
Figure S17. EXCSY spectrum of DCP-(L-Cys)$_2$ in the presence of YCl_3
Table S4. Nucleotide sequences of the genes of ERp29 with TEV site preceding a C-terminal His_6 tag, GB1 preceded by a MASMTG tag and followed by a TEV site and C-terminal His_6 tag, and list of mutation primers used
References

Synthesis of (*S*)-3-amino-4-mercaptobutanoic acid hydrochloride (β-cysteine, 5)
A synthesis of β-cysteine was described by Birkofer and Birkofer (1956) without spectroscopic characterisation data of the final compound. Below is a detailed description of the synthetic route chosen in the present work.

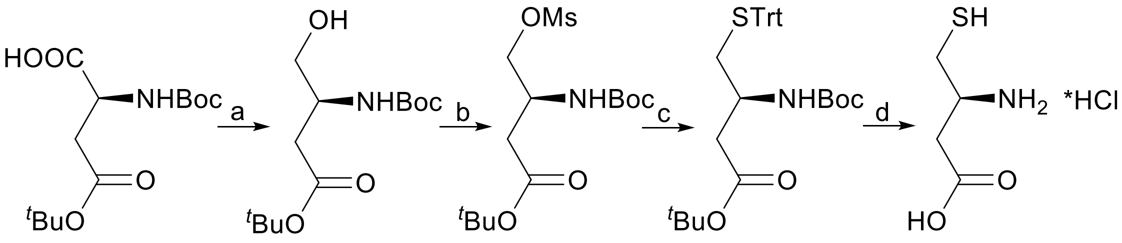

1 **2** **3** **4** **5**

Reagents and conditions: **a)** *N*-methyl morpholine (1.3 equiv), ethyl chloroformate (1.2 equiv), THF, 0 °C, 30 min. then $NaBH_4$ (2 equiv) in water, 0 °C, 30 min, 73 %; **b)** DIPEA (1.7 equiv), methanesulfonyl chloride (1.2 equiv), toluene, 0 °C, 2 h, 85 %; **c)** triphenylmethanethiol (1.8 equiv), KO^{t}Bu (1.6 equiv), THF, 0 °C, 30 min., 71 %; **d)** triethylsilane/TFA, DCM, 5 h, then anhydrous HCl/Et_2O, 71 %.

***tert*-Butyl (*S*)-3-((*tert*-butoxycarbonyl)amino)-4-hydroxybutanoate (2)**

Ethyl chloroformate (1.90 mL; 20 mmol) was added dropwise to a cooled (0 °C) solution of Boc-*L*-aspartic acid 4-*tert*-butyl ester (**1**) (5.00 g; 17.3 mmol) and *N*-methyl morpholine (2.40 mL; 22.0 mmol) in THF (150 mL). The resulting mixture was stirred at 0 °C for 30 minutes when it was pured into a cooled solution of $NaBH_4$ (1.32 g; 35.0 mmol) in water (50 mL) with intensive stirring. The resulting emulsion was stirred intensively for 30 minutes and THF was subsequently removed under reduced pressure. The residue was partitioned between brine (100 mL) and EtOAc (200 mL). The organic phase was washed with aq $KHSO_4$ (5 %; 2 x 100 mL), saturated aq $NaHCO_3$ (100 mL) and brine (100 mL) and evaporated under reduced pressure. The crude product was purified by flash column chromatography (mobile phase hexanes/EtOAc with gradient from 4/1 to 1/1). 3.46 g (73 %) of colourless amorphous solid was obtained. For ^{1}H-NMR data, see Bergeron et al. (1998).

***tert*-Butyl (*S*)-3-((*tert*-butoxycarbonyl)amino)-4-((methylsulfonyl)oxy)butanoate (3)**

Methanesulfonyl chloride (1.1 mL; 14 mmol) was added dropwise to a cooled (0 °C) solution of *tert*-butyl (*S*)-3-((*tert*-butoxycarbonyl)amino)-4-hydroxybutanoate (**2**) (3.25 g; 11.8 mmol) and DIPEA (3.5 mL; 20 mmol) in toluene (150 mL). The reaction was stirred at 0 °C for 2

hours. TLC analysis showed complete conversion. The reaction mixture was partitioned between aq $KHSO_4$ (5 %; 150 mL) and EtOAc (150 mL). The organic phase was washed with aq $KHSO_4$ (5 %; 100 mL), saturated aq $NaHCO_3$ (100 mL) and brine (100 mL) and evaporated under reduced pressure. The residue was purified by flash column chromatography (mobile phase hexanes/EtOAc with gradient from 4/1 to 1/1). 3.56 g (85 %) of yellowish oil was obtained. For ^{1}H-NMR data, see Kim et al. (2014).

<u>*tert*-Butyl (*S*)-3-((*tert*-butoxycarbonyl)amino)-4-(tritylthio)butanoate (4)</u>

Potassium *tert*-butoxide (1.7g; 15 mmol) was added portionwise to a cooled (-20 °C) solution of triphenylmethanethiol (4.7 g; 17 mmol) in dry THF (150 mL). The reaction mixture was stirred until all solids dissolved and subsequently slowly cannulated into a cooled solution (0 °C) of *tert*-butyl (*S*)-3-((*tert*-butoxycarbonyl)amino)-4-((methylsulfonyl)oxy)butanoate (**3**) (3.40 g; 9.62 mmol) in dry THF (50 mL). The resulting solution was stirred at 0 °C for about 30 minutes. At this point TLC analysis indicated complete conversion of **3**. The reaction mixture was quenched by the addition of saturated aq $NaHCO_3$ (100 mL) and THF was removed under reduced pressure. The residue was partitioned between brine (100 mL) and EtOAc (200 mL). The organic phase was washed with saturated aq $NaHCO_3$ (100 mL) and brine (100 mL) and evaporated under reduced pressure. The crude product was purified by flash column chromatography (mobile phase hexanes/EtOAc with gradient from 20/1 to 4/1). 3.62 g (71 %) of white foam was obtained.

<u>(*S*)-3-Amino-4-mercaptobutanoic acid hydrochloride (5)</u>

Trifluoroacetic acid (8 mL) was added to a cooled (0 °C) solution of *tert*-butyl (*S*)-3-((*tert*-butoxycarbonyl)amino)-4-(tritylthio)butanoate (**4**) (3.60 g; 6.75 mmol) and triethylsilane (5 mL) in DCM (30 mL). The reaction mixture was stirred at ambient temperature 5 h. LC-MS analysis indicated complete conversion. The volatiles were removed under reduced pressure. The residue was dissolved in EtOAc (50 mL) and anhydrous HCl (2 M in ether; 6 mL) was added. The volatiles were removed under reduced pressure and the HCl treatment was repeated 3 times. The glue-like residue was suspended in EtOAc (30 mL) and sonicated 30 minutes which promoted crystallization of the amorphous substance. The reaction product was collected by centrifugation and it was washed with additional EtOAc (2 x 5 mL). After drying under vacuum 1.05 g (91 %) of white microcrystalline solid was obtained.

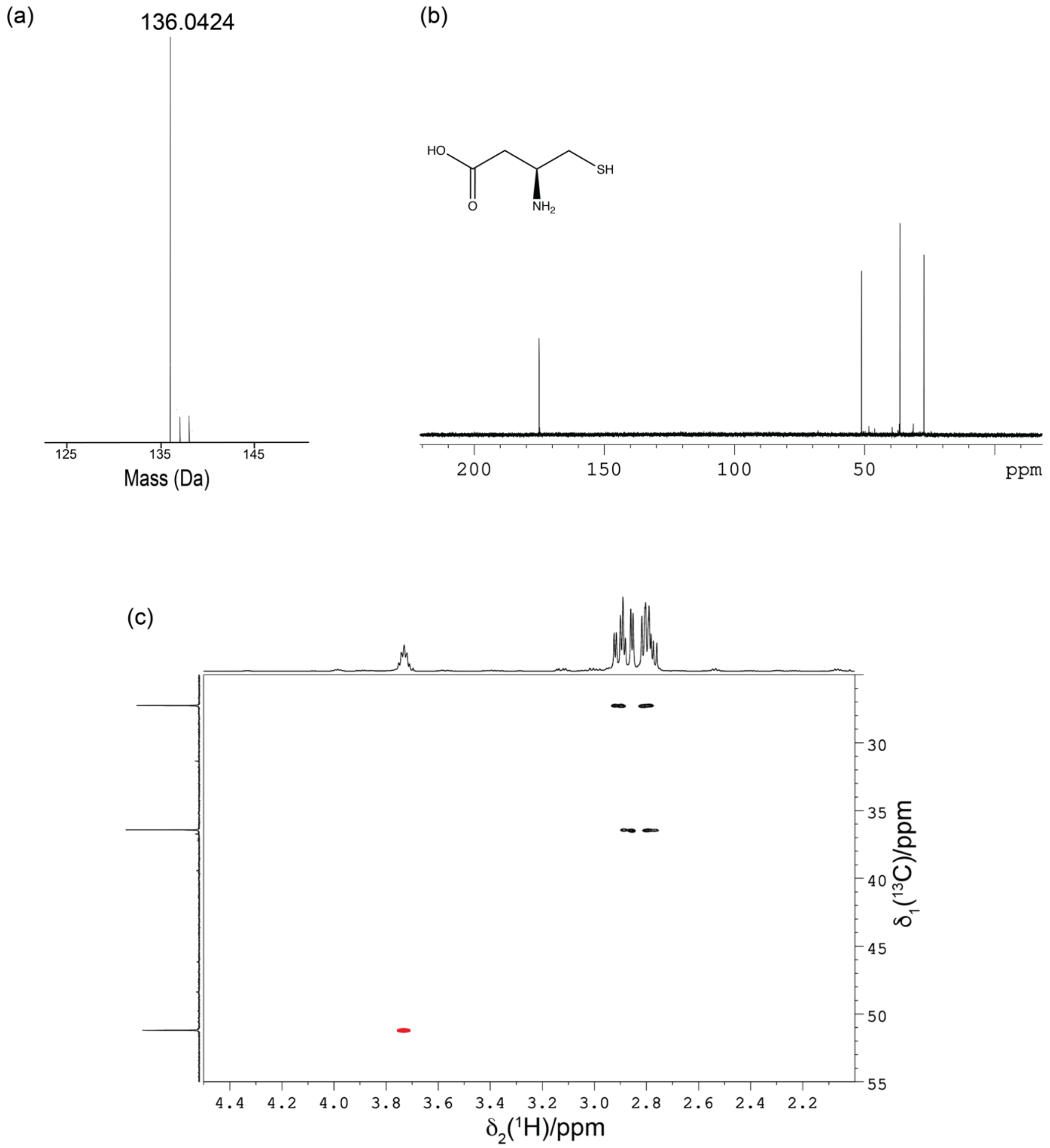

Figure S1. Mass spectrometric and NMR analysis of (*S*)-3-amino-4-mercaptobutanoic acid (β-cysteine). (a) HR-ESI mass spectrum of β-cysteine. The calculated mass is 135.18 Da. (b) and (c) show, respectively, the 1D ^{13}C-NMR spectrum and [^{13}C,^{1}H]-HSQC spectrum recorded of a 5 mM sample of β-cysteine prepared in D_2O.

Synthesis of DCP-(L-Cys)₂

2,6-pyridinedicarbonitrile (41.0 mg, 0.317 mmol) and L-cysteine hydrochloride (100 mg, 0.634 mmol) were added to 2 ml MeOH/water (1:1). The pH was adjusted to 7 with KOH and the mixture was stirred for 3 days. The solvent was removed under reduced pressure and the crude product was purified using a 10 g silica gel cartridge (5–20 % water:EtOH) to yield the title compound (44.2 mg, 41 %) as an off-white crystalline powder.

LRMS (ESI) *m/z*: 338.0 $[M+H]^+$

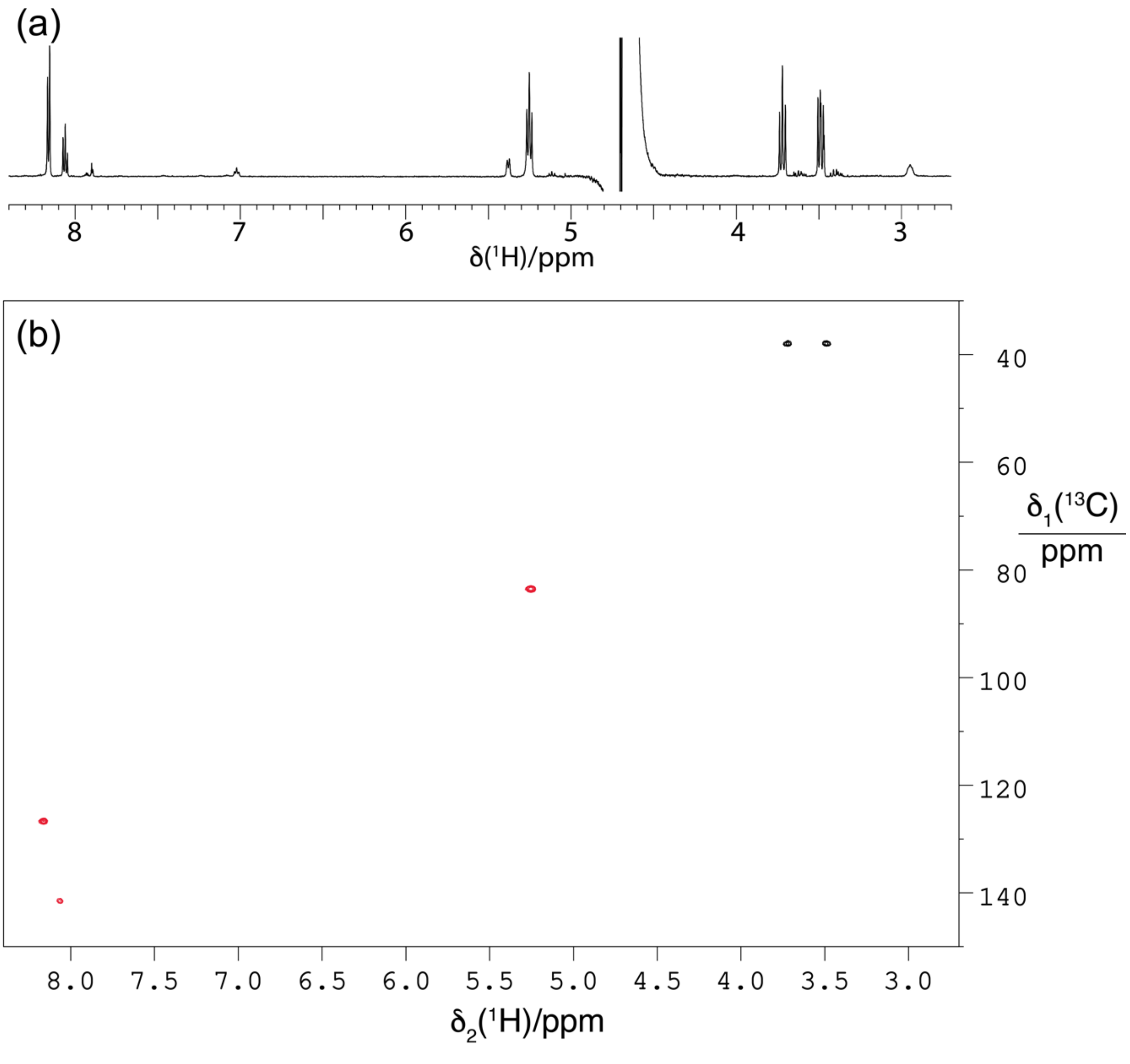

Figure S2. NMR spectra of 0.6 mM DCP-(L-Cys)$_2$ in D_2O at 25 °C. Spectra recorded on a 600 MHz NMR spectrometer. (a) ^{1}H-NMR spectrum with presaturation of the HDO signal. Small signals at about 7, 5.4 and 2.9 ppm are from a minor conformer that is in chemical exchange with the main species. (b) [^{13}C,^{1}H]-HSQC spectrum. Positive (black) and negative (red) cross-peaks are from CH_2 and CH groups, respectively.

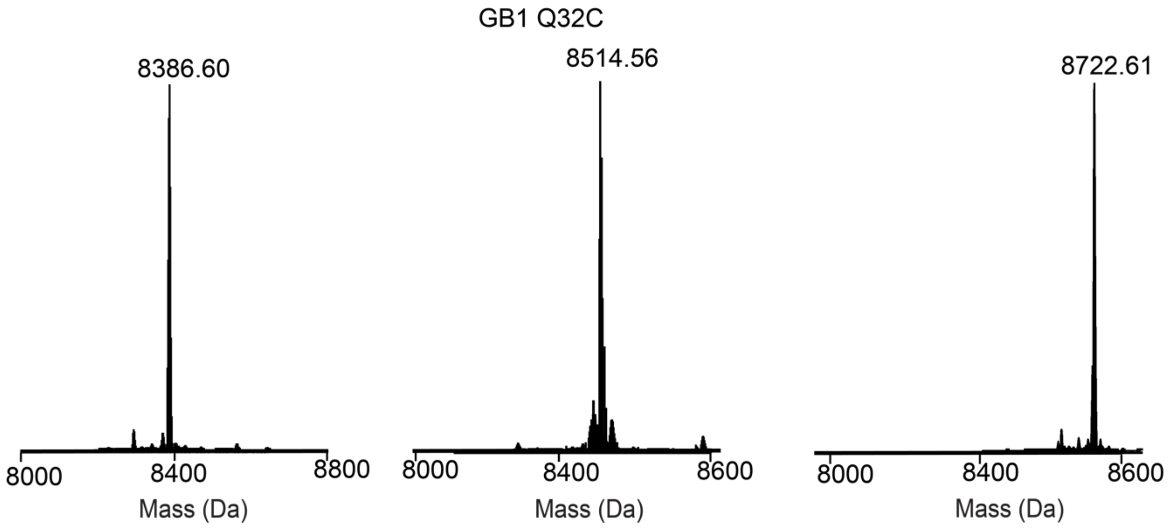

Figure S3. Deconvoluted intact protein mass spectra of uniformly ^{15}N-labelled GB1 Q32C. Left panel: before tagging reaction with FDCP. Centre panel: after reaction with FDCP. Right panel: after the complete assembly of the DCP-(L-Cys)$_2$ tag on the protein (expected mass increase 336 Da).

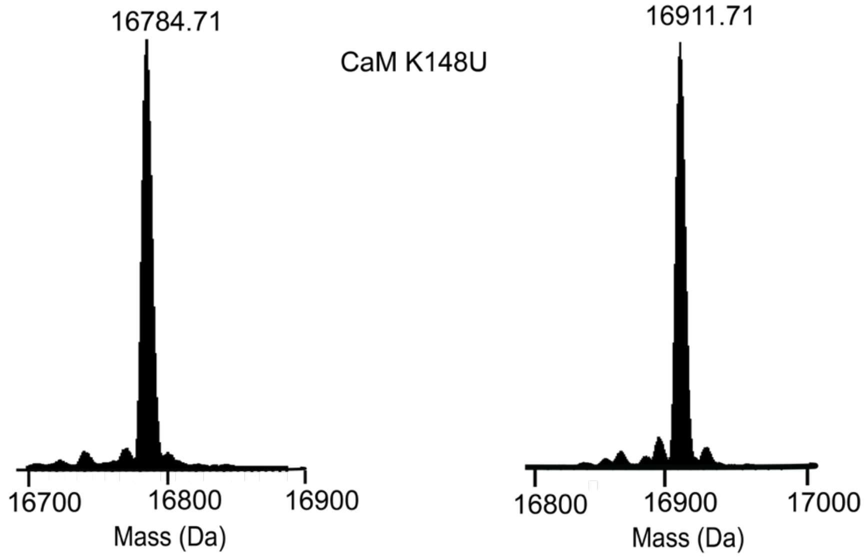

Figure S4. Deconvoluted intact protein mass spectra of calmodulin K148U. Left panel: before reaction with FDCP. The calculated mass is 16785.30 Da. Right panel: after the reaction. The reaction was carried out at 25 °C for 10 minutes. The expected mass increase is 128 Da.

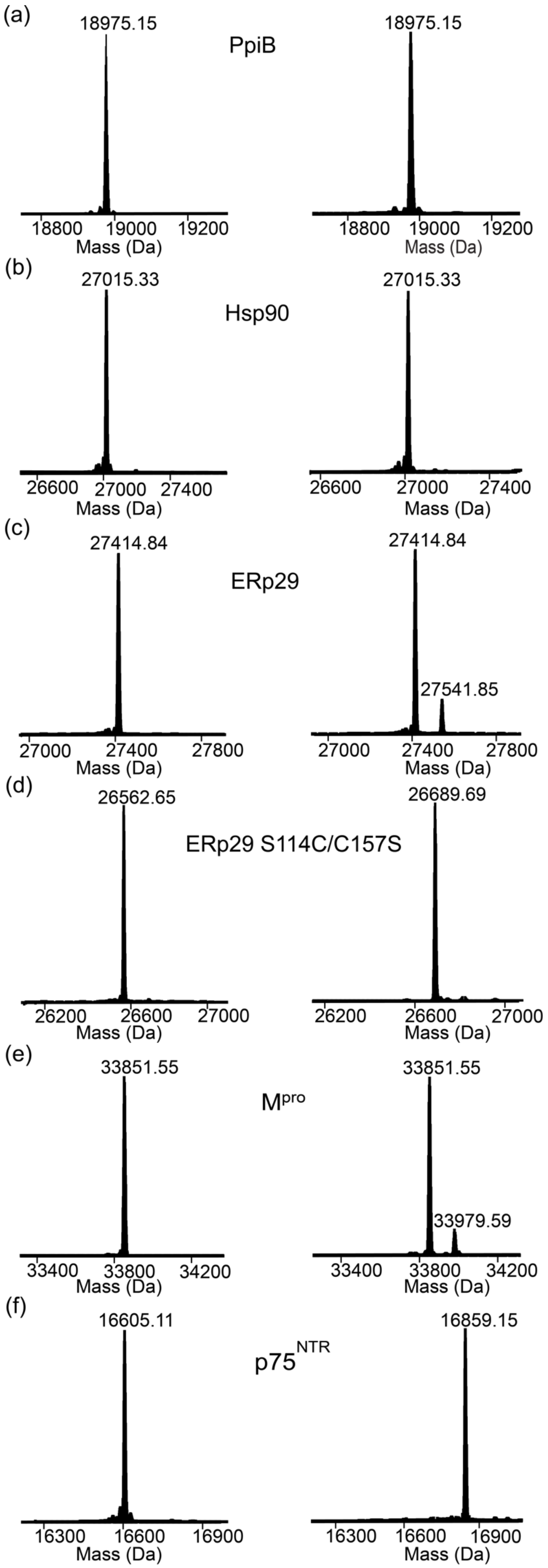

(a)
18975.15
PpiB
18975.15
18800 19000 19200
Mass (Da)
18800 19000 19200
Mass (Da)
(b)
27015.33
Hsp90
27015.33
26600 27000 27400
Mass (Da)
26600 27000 27400
Mass (Da)
(c)
27414.84
ERp29
27414.84
27541.85
27000 27400 27800
Mass (Da)
27000 27400 27800
Mass (Da)
(d)
26562.65
ERp29 S114C/C157S
26689.69
26200 26600 27000
Mass (Da)
26200 26600 27000
Mass (Da)
(e)
33851.55
Mpro
33851.55
33979.59
33400 33800 34200
Mass (Da)
33400 33800 34200
Mass (Da)
(f)
16605.11
p75NTR
16859.15
16300 16600 16900
Mass (Da)
16300 16600 16900
Mass (Da)

Figure S5. Deconvoluted intact protein mass spectra illustrating the reactivity of the FDCP tag with cysteine residues of different solvent exposure. The spectra in the left and right panels show the whole-protein mass spectra before and after reaction with the tag, respectively. The expected mass increase upon addition of a single DCP tag is 128 Da. (a) *E. coli* PpiB. The protein contains two buried cysteine residues. The calculated mass of the untagged protein is 18976.32 Da. (b) N-terminal domain of *P. falciparum* Hsp90. The protein contains a single cysteine residue with limited solvent exposure. Calculated mass (without tag): 27016.40 Da. (c) Rat ERp29. The protein contains a single cysteine residue with partial solvent exposure. Calculated mass (without tag): 27415.20 Da. (d) Rat ERp29 G147C/C157S. The protein contains one highly solvent-exposed cysteine residue. Calculated mass (without tag): 26563.28 Da. (e) SARS-2 main protease (M^{pro}). The protein contains 12 cysteine residues, three of which are partially solvent exposed (including the active-site residue C145). Calculated mass (without tag): 33851.55 Da. (f) Intracellular domain of $p75^{NTR}$. The protein contains two highly solvent-exposed cysteine residues. Calculated mass (without tag): 16606.23 Da.

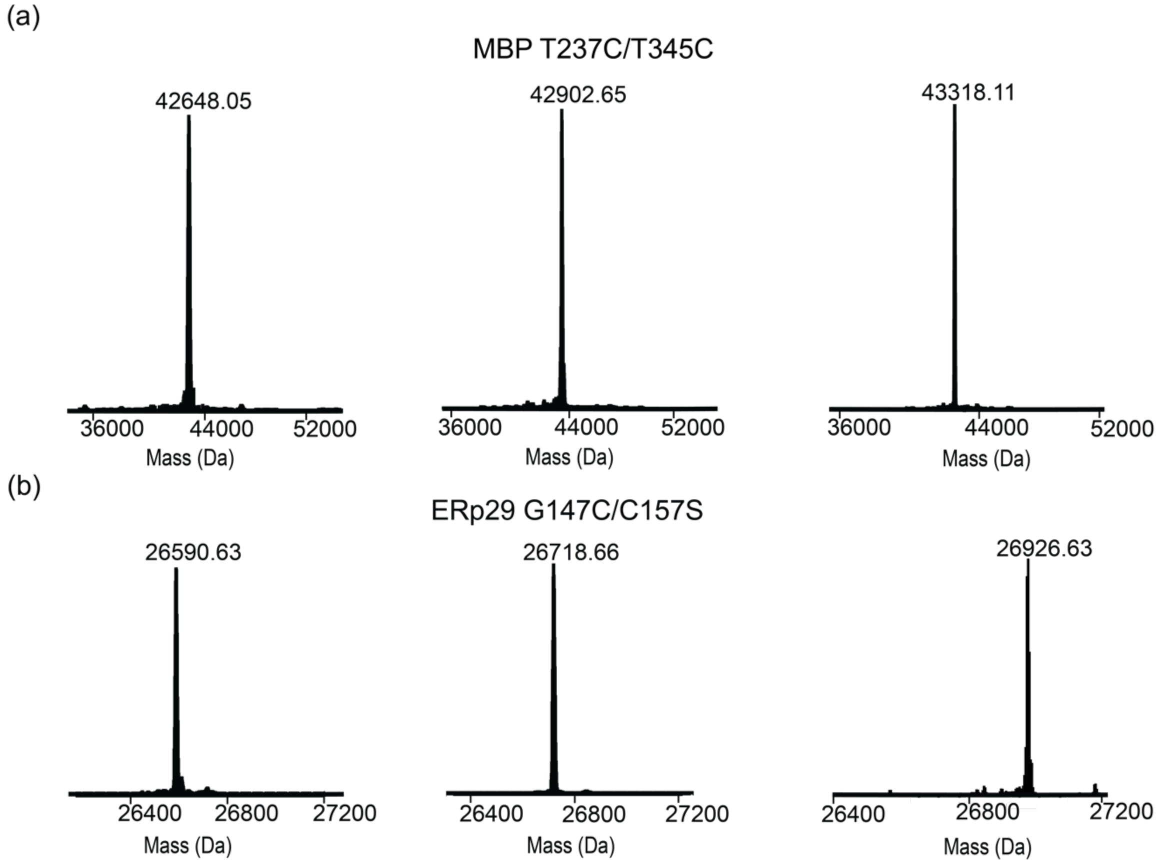

Figure S6. Deconvoluted intact protein mass spectra showing the reaction of the FDCP tag with cysteine residues in the protein (expected mass increase per DCP tag is 128 Da) and subsequent reaction of each DCP tag with two cysteine molecules (expected mass increase per DCP tag is 208 Da). Left panel: before reaction with the tag. Calculated masses are 42648.53 and 26591.96 Da respectively. Centre panel: after reaction with the FDCP tag. Right panel: after reaction with excess free cysteine to complete the metal binding tag. (a) MBP T237C/T345C. (b) ERp29 G147C/C157S.

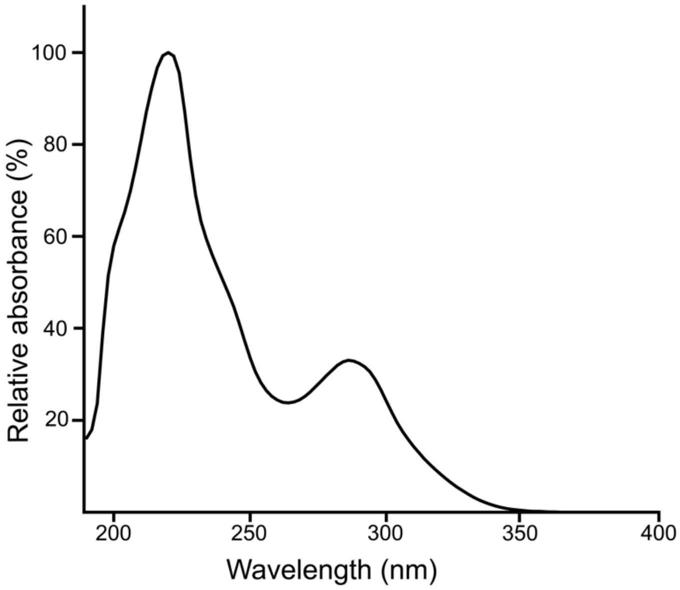

Figure S7. UV/Vis absorption spectrum of DCP-(L-Cys)$_2$. The spectrum was recorded of the DCP-(L-Cys)$_2$ fraction during a HPLC-MS run, using an Agilent mass spectrometer equipped with a reverse-phase column, a gradient from 5 % MeOH:water to 90 % MeOH:water in the presence of 0.1 % TFA and a temperature of 30 °C. Using a separate sample of DCP reacted with excess L-cysteine, the molar extinction coefficient ε at 280 nm was determined to be 6850 M^{-1} cm^{-1}. A sample of DCP reacted in the same way with excess L-penicillamine yielded ε_{280} = 5400 M^{-1} cm^{-1}.

Table S1. PCSs of backbone amide protons generated with $TbCl_3$ and $TmCl_3$ in GB1 Q32C with DCP-$(Cys)_2$ tags of different chirality.

GB1-DCP-(L-Cys)$_2$				GB1-DCP-(D-Cys)$_2$			
Tb^{3+}		Tm^{3+}		Tb^{3+}		Tm^{3+}	
Residue	PCS^{exp}/ppm	Residue	PCS^{exp}/ppm	Residue	PCS^{exp}/ppm	Residue	PCS^{exp}/ppm
Thr2	0.500	Tyr3	0.420	Thr2	-0.233	Thr2	0.118
Lys4	0.726	Lys4	0.329	Tyr3	-0.408	Tyr3	0.198
Leu5	0.905	Leu5	0.442	Lys4	-0.291	Lys4	0.147
Ile6	0.538	Ile6	0.298	Leu5	-0.423	Leu5	0.188
Leu7	0.489	Leu7	0.302	Ile6	-0.325	Ile6	0.145
Asn8	0.188	Asn8	0.162	Leu7	-0.374	Leu7	0.153
Gly9	0.093	Gly9	0.125	Asn8	-0.297	Asn8	0.115
Gly14	0.350	Gly14	0.248	Gly9	-0.285	Gly9	0.104
Glu15	0.727	Glu15	0.408	Lys10	-0.202	Lys10	0.049
Val21	1.469	Thr16	0.517	Thr11	-0.207	Thr11	0.065
Asp22	1.221	Thr18	0.423	Leu12	-0.267	Leu12	0.091
Ala23	0.713	Ala20	0.671	Lys13	-0.274	Lys13	0.102
Thr44	0.205	Val21	1.091	Gly14	-0.334	Gly14	0.129
Tyr45	0.246	Ala26	1.025	Thr16	-0.443	Thr16	0.193
Asp46	0.380	Glu27	0.697	Thr17	-0.296	Thr17	0.143
Asp47	0.308	Lys28	0.694	Thr18	-0.464	Thr18	0.210
Ala48	0.286	Thr44	0.117	Ala20	-0.605	Ala20	0.316
Thr49	0.319	Tyr45	0.109	Val21	-0.638	Val21	0.448
Lys50	0.409	Asp46	0.167	Thr25	-0.227	Thr25	0.113
Thr51	0.459	Asp47	0.128	Phe30	-1.376	Phe30	0.540
Thr53	0.348	Ala48	0.118	Thr44	-0.103	Thr44	0.048
Val54	0.274	Thr49	0.137	Thr49	-0.090	Asp46	0.060
Thr55	0.056	Lys50	0.175	Asp46	-0.097	Thr49	0.045
		Thr51	0.202	Lys50	-0.108	Lys50	0.067
		Phe52	0.300	Thr51	-0.140	Thr51	0.078
		Thr53	0.180	Thr53	-0.176	Thr53	0.083
		Val54	0.197	Val54	-0.293	Val54	0.118
		Thr55	0.076	Thr55	-0.171	Thr55	0.055
				Glu56	-0.204	Glu56	0.109

Table S2. PCSs of backbone amide protons generated with $TbCl_3$ and $TmCl_3$ in GB1 Q32C with DCP tags reacted with penicillamines of different chirality.

GB1-DCP-(L-pen)$_2$				GB1-FDCP-(D-pen)$_2$			
Tb^{3+}		Tm^{3+}		Tb^{3+}		Tm^{3+}	
Residue	PCS^{exp}/ppm	Residue	PCS^{exp}/ppm	Residue	PCS^{exp}/ppm	Residue	PCS^{exp}/ppm
Met1	1.622	Met1	-0.787	Thr2	-0.856	Thr2	0.478
Thr2	1.033	Thr2	-0.519	Lys4	-0.845	Lys4	0.471
Tyr3	1.505	Tyr3	-0.748	Leu5	-1.065	Leu5	0.570
Lys4	0.935	Lys4	-0.455	Ile6	-0.696	Ile6	0.347
Ile6	0.429	Ile6	-0.220	Leu7	-0.686	Leu7	0.328
Leu7	0.289	Leu7	-0.197	Asn8	-0.507	Asn8	0.223
Glu15	0.387	Glu15	-0.281	Gly9	-0.432	Gly9	0.180
Thr18	1.436	Thr18	-0.752	Lys10	-0.297	Lys10	0.122
Gly41	-0.409	Thr25	-0.505	Thr11	-0.279	Thr11	0.120
Trp43	-0.250	Gly41	0.199	Leu12	-0.351	Leu12	0.137
Tyr45	0.159	Trp43	0.127	Lys13	-0.401	Lys13	0.155
Asp46	0.398	Asp46	-0.162	Gly14	-0.566	Gly14	0.249
Asp47	0.345	Asp47	-0.521	Glu15	-0.728	Glu15	0.331
Ala48	0.352	Ala48	-0.153	Thr16	-1.052	Thr16	0.516
Thr49	0.407	Thr49	-0.186	Thr18	-1.504	Thr25	0.421
Lys50	0.554	Lys50	-0.254	Thr25	-0.771	Gly41	0.117
Thr51	0.554	Thr51	-0.254	Gly41	-0.337	Asp46	0.145
Phe52	0.650	Phe52	-0.300	Asp46	-0.218	Asp47	0.074
Thr53	0.235	Thr53	-0.101	Asp47	-0.068	Ala48	0.099
				Ala48	-0.118	Thr49	0.137
				Thr49	-0.221	Lys50	0.188
				Lys50	-0.276	Thr51	0.219
				Thr51	-0.358	Phe52	0.350
				Phe52	-0.633	Thr53	0.188
				Thr53	-0.350	Glu56	0.129
				Val54	-0.529		
				Thr55	-0.190		
				Glu56	-0.315		

Table S3. $^{1}D_{HN}$ RDCs of backbone amides of GB1 Q32C with different DCP tags loaded with Tb^{3+} ions.

DCP-(D-pen)$_2$-Tb		DCP-(L-Cys)$_2$-Tb	
Residue	RDC/Hz	Residue	RDC/Hz
Thr2	11.1	Thr2	-6.6
Leu5	-11.3	Lys4	-6.8
Ile6	-18.7	Leu5	5.7
Leu7	-9.5	Ile6	2.0
Asn8	-11.1	Leu7	5.6
Gly9	-5.7	Asn8	3.9
Lys10	5.3	Gly9	6.7
Thr11	2.0	Lys10	2.3
Leu12	-8.0	Gly14	0.2
Lys13	-14.6	Glu15	-6.2
Gly14	-12.1	Val21	2.0
Glu15	-13.4	Thr44	8.3
Thr16	-17.5	Tyr45	-1.1
Thr25	-2.3	Asp46	4.8
Gly41	10.0	Ala48	9.2
Asp46	-13.3	Thr49	-1.0
Asp47	-10.0	Lys50	-5.6
Ala48	1.5	Thr51	-0.1
Thr49	6.0	Phe52	5.1
Lys50	-14.3	Thr53	5.1
Thr51	-9.2	Thr55	5.1
Phe52	-12.5		
Thr53	-13.9		
Val54	-9.9		
Thr55	-9.9		
Glu56	-2.9		

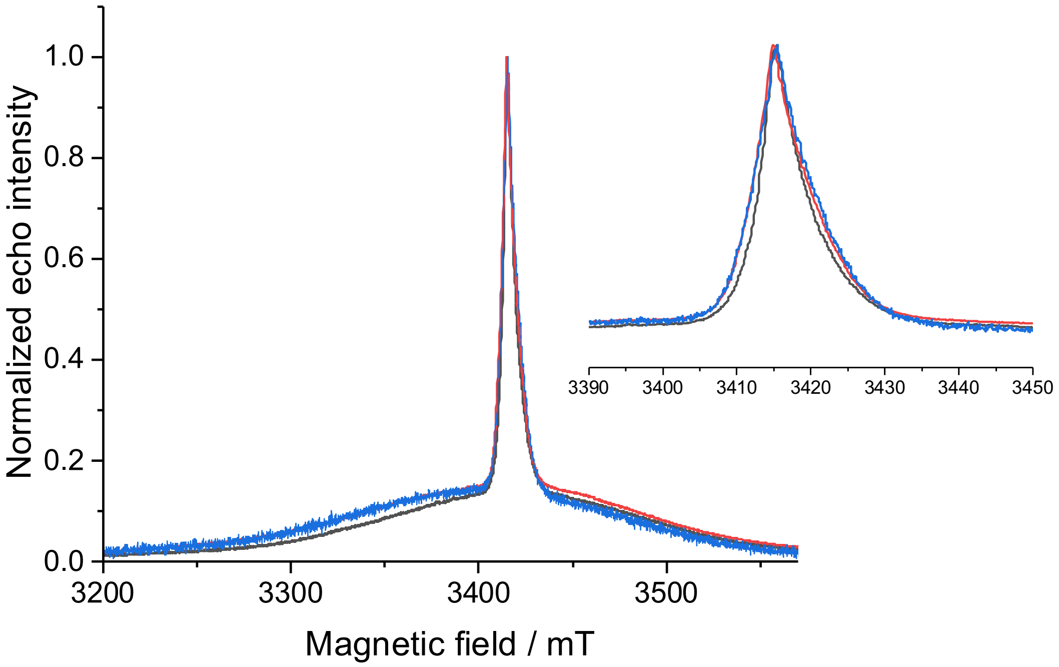

Figure S8. Echo-detected W-band EPR spectra of ERp29 S114/C157S DCP-(β-Cys)$_2$-Gd (black), MBP T237U/T345U DCP-(L-Cys)$_2$-Gd (red) and MBP T237C/T345C DCP-(L-Cys)$_2$-Gd (blue). All samples targeted a metal-to-tag ratio of 1:1 but contained about 20 % excess of Gd^{3+} ions because the protein concentration was overestimated due to neglecting the contribution of the tag to the absorbance at 280 nm.

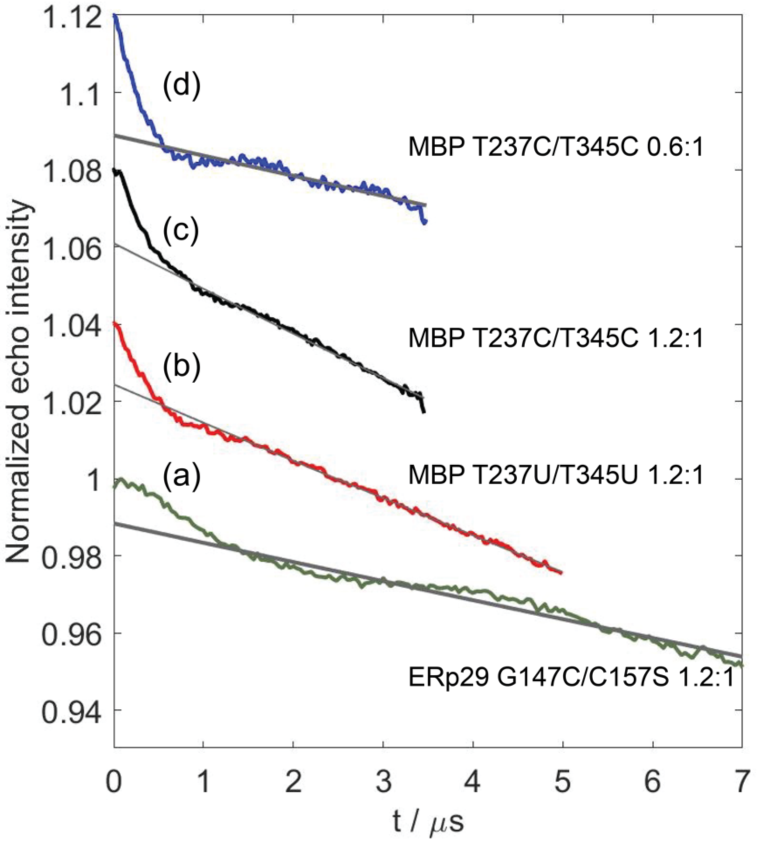

Figure S9. Primary DEER traces of Fig. 4 in the main text. The background decay is shown as grey lines. (a) ERp29 G147C/C157S with 1.2:1 metal-to-tag ratio. (b) MBP T237U/T345U with a metal-to-tag ratio of 1.2:1. (c) MBP T237C/T345C with a metal-to-tag ratio of 1.2:1. (d) Same as (c), but with a metal-to-tag ratio of 0.6:1.

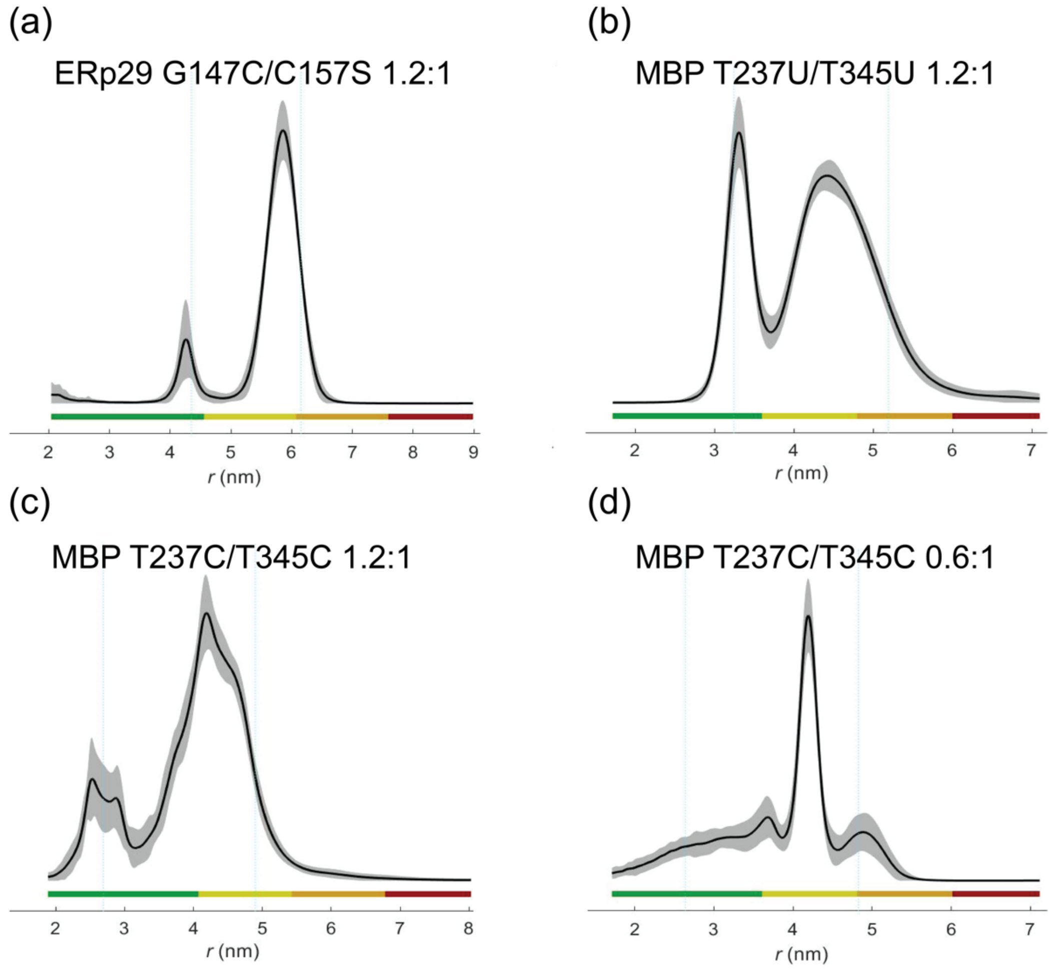

Figure S10. Distance distributions of Fig. 4 analysed by DeerNet as implemented in DeerAnalysis2022 (Worswick et al., 2018), with colour coding of the reliability regions as defined in DeerAnalysis (Jeschke et al., 2006), corresponding to the DEER evolution time used (green: the shape of the distance distribution is reliable. Yellow: the mean distance and distribution width are reliable. Orange: the mean distance is reliable. Red: long-range distance contributions may be detectable but cannot be quantified). The solid lines represent the distributions with the best r.m.s.d. from the experimental data and the striped regions represent the variation of alternative distributions (±2 times the standard deviation) obtained by varying the parameters of the background correction and noise as calculated by the validation tool in the DeerAnalysis software package. The parameter ranges used for the validations were the default ones: white noise 0–1.5, background start $0.2*t_{max}$–$0.6*t_{max}$, and background dimension 3–3.6.

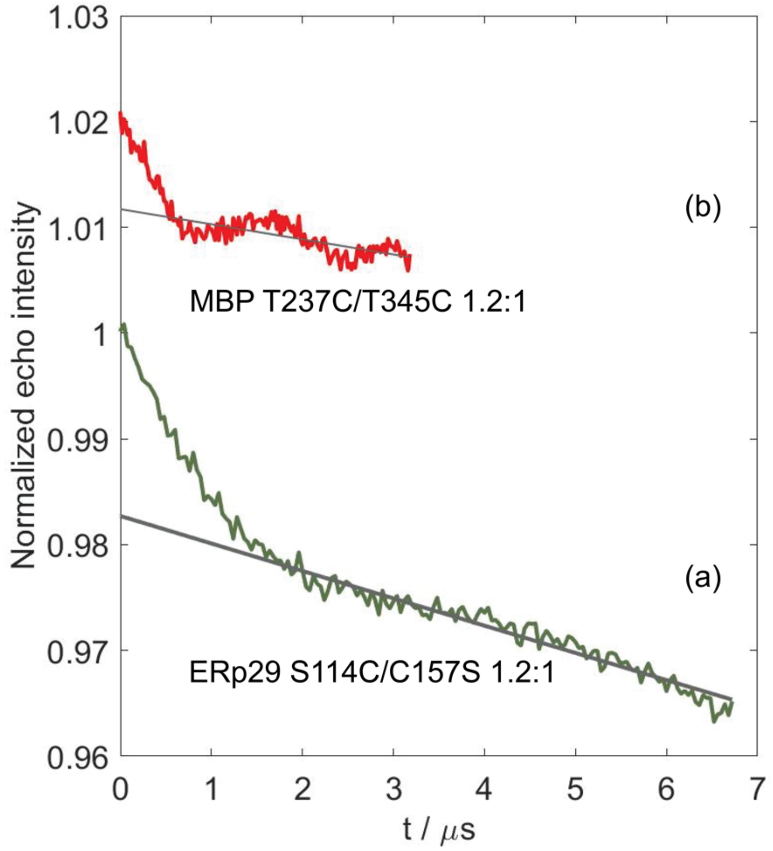

Figure S11. Raw DEER traces of Fig. 5 in the main text. The background decay is shown as grey lines. (a) ERp29 S114C/C157S. (b) MBP T237C/T345C.

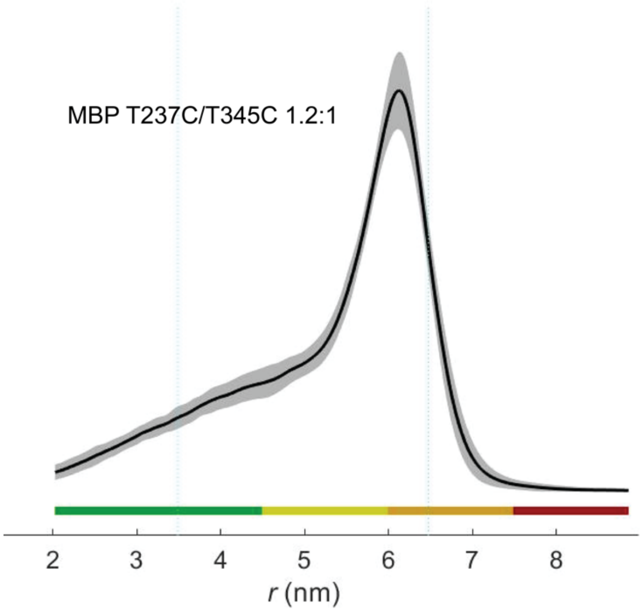

Figure S12. Distance distribution of MBP T237C/T345C (Fig. 5 of the main text) analysed by DeerNet (Worswick et al., 2018) with colour coding of the reliability regions as defined in Fig. S10.

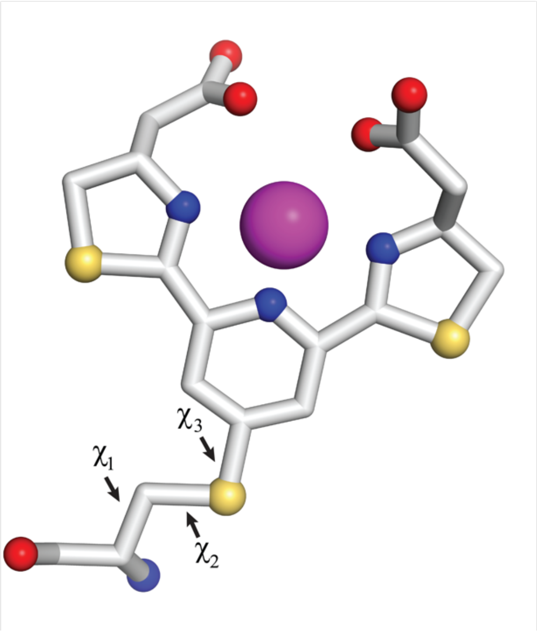

Figure S13. Conformation of the DCP-(β-Cys)$_2$-Gd tag attached to cysteine as used for modelling distance distributions. Dihedral angles χ of rotatable bonds are labelled. Blue, red, yellow and magenta balls identify atoms of nitrogen, oxygen, sulfur and gadolinium, respectively. The conformation was modelled using ChemDraw, which placed the metal ion 1.9 Å from the pyridine nitrogen and 2.3 Å from the thiazoline nitrogens.

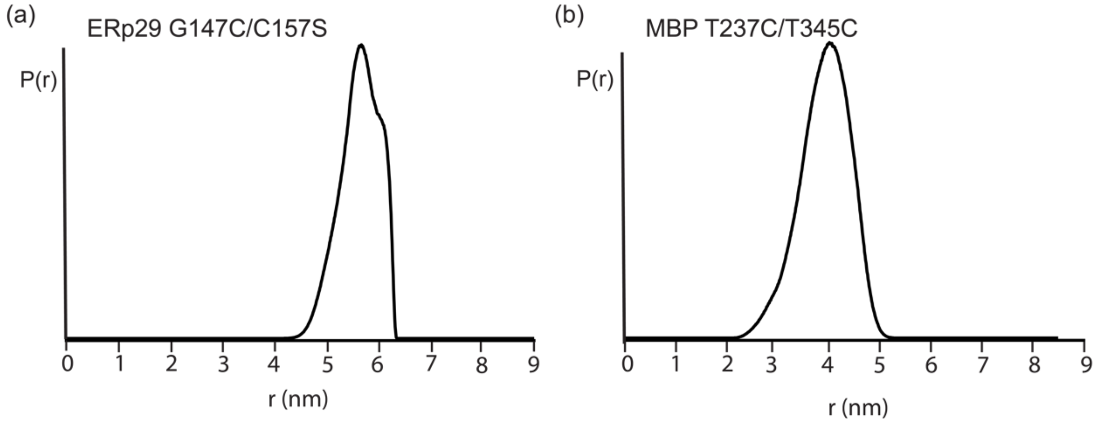

Figure S14. Gd^{3+}–Gd^{3+} distance distributions calculated with the program PyParaTools (Stanton-Cook et al., 2014) using rotamer libraries of the DCP-Cys_2-Gd tag bound to cysteine, generated by varying the χ_1 angle by ±30° around the staggered rotamers and allowing free rotation about the χ_2 and χ_3 angles. (a) ERp29 G147C/C157S. (b) MBP T237C/T345C.

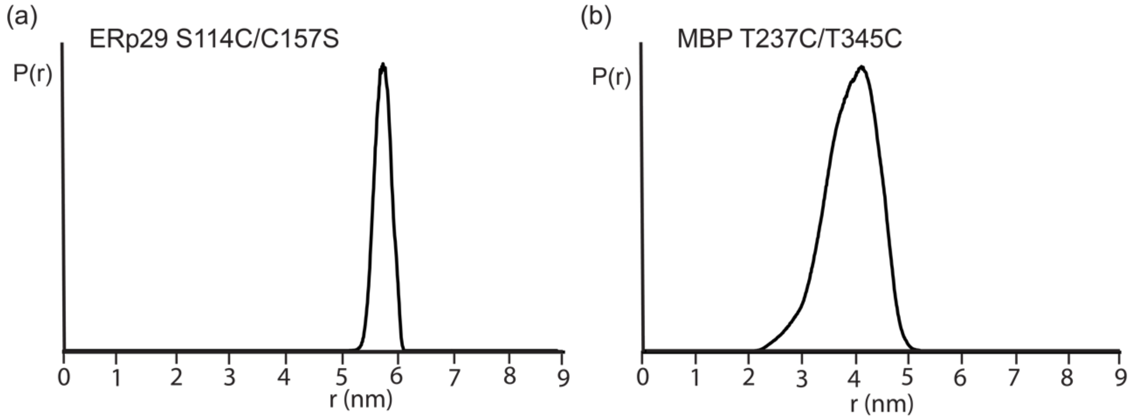

Figure S15. Gd^{3+}–Gd^{3+} distance distributions calculated with the program PyParaTools using rotamer libraries of the DCP-(β-Cys)$_2$-Gd tag bound to cysteine (see Fig. S13), generated by varying the χ_1 angle by ±30° around the staggered rotamers and allowing free rotation about the χ_2 and χ_3 angles. (a) ERp29 S114C/C157S. (b) MBP T237C/T345.

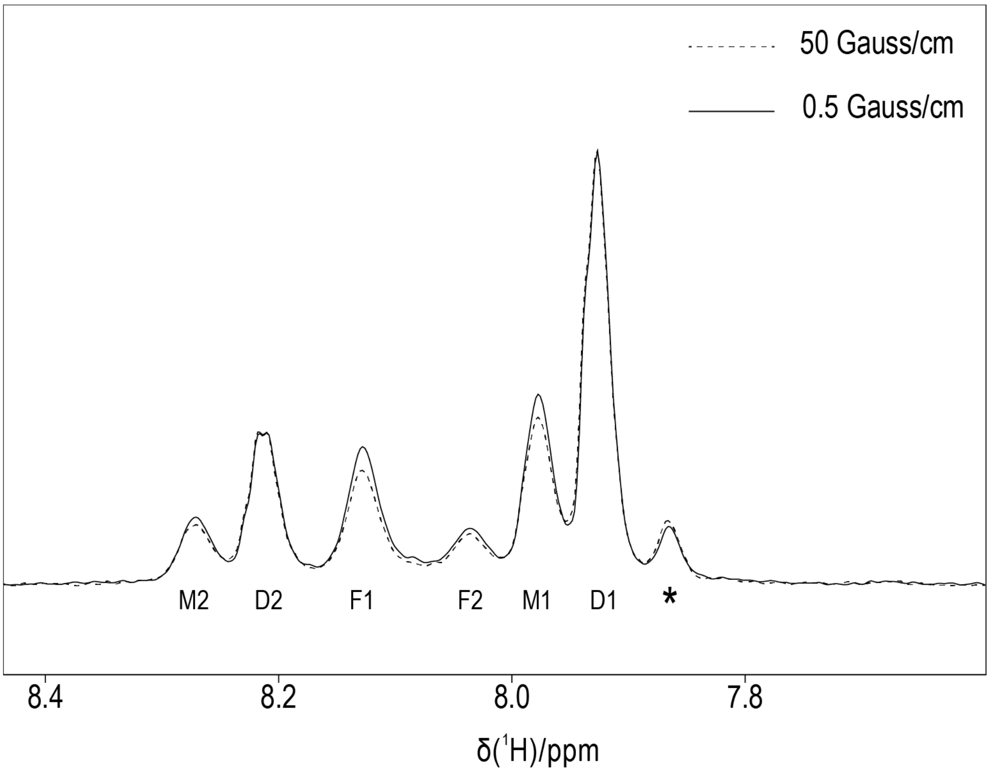

Figure S16. Diffusion experiment of DCP-(L-Cys)$_2$ in the presence of YCl_3. The spectrum was recorded of a 0.3 mM solution of DCP-(L-Cys)$_2$ in 10 mM HEPES buffer (pH 7) in D_2O with 0.5 equivalents of YCl_3, using a 800 MHz NMR spectrometer. The pulse sequence used a stimulated echo with bipolar gradients (Bruker pulse program stebpggp1s191d). Each pulsed field gradient was 2 ms and the duration of the diffusion delay Δ was limited to 10 ms to prevent chemical exchange from equilibrating the peak intensities. The spectra plotted with solid and dashed lines were recorded with weak (0.5 Gauss/cm) and strong (50 Gauss/cm) gradients, the latter resulting in about 11-fold signal attenuation. The loss in sensitivity was compensated by recording the attenuated spectrum with 8192 instead of 1024 scans. Scaling of the peaks D1 and D2 for closest superimposition, the signals of the free ligand (F1 and F2) and the 1:1 complex (M1 and M2) are smaller with strong than weak gradients, indicating more rapid diffusion as expected, if D1 and D2 correspond to the 2:1 ligand-to-protein complex. The signal labelled with a star showed no exchange cross-peaks (Fig. S17) and is unassigned. A corresponding diffusion experiment conducted with GB1 Q32C tagged with DCP-(L-Cys)$_2$ and titrated with 0.6 equivalents $TmCl_3$ yielded no measurable difference in peak attenuation between diamagnetic and paramagnetic signals.

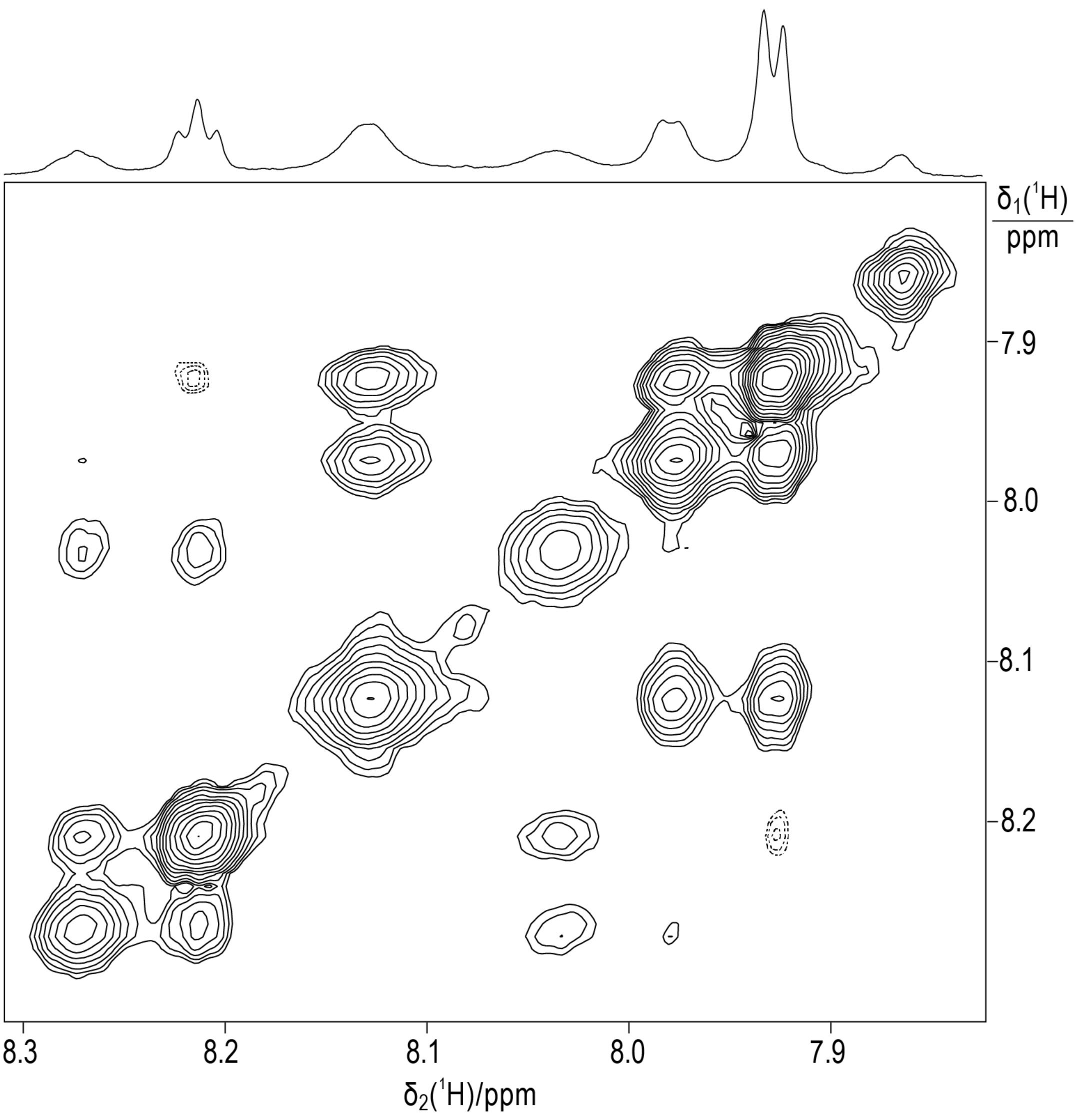

Figure S17. EXCSY spectrum of DCP-(L-Cys)$_2$ in the presence of YCl_3. The spectrum was recorded of the sample used to record the data of Fig. S16. The 1D NMR spectrum (see Fig. 6 of the main text) is shown at the top. The spectrum was recorded at 800 MHz with a mixing time of 25 ms, t_{1max} = 40 ms, t_{2max} = 160 ms. Negative contour levels associated with zero-quantum cross-peaks are plotted with dashed lines. Dividing the cross-peak intensities by the diagonal intensities and the mixing time yields an exchange rate between free and bound metal ion of about 10 s^{-1}.

Table S4. Nucleotide sequences of the genes of ERp29 with TEV site preceding a C-terminal His_6 tag, GB1 preceded by a MASMTG tag and followed by a TEV site and C-terminal His_6 tag, and list of mutation primers used.

Protein	DNA Sequence	Primers
ERp29_TEV_C-His_6	ATGCTGCACACGAAGGGCGCCCTTCCCTTGGA<br>CACAGTCACTTTCTACAAGGTCATTCCCAAAAG<br>CAAGTTCGTCTTGGTGAAGTTCGACACCCAGTA<br>CCCCTATGGAGAGAAGCAAGATGAGTTTAAGC<br>GTCTGGCTGAGAACTCAGCCTCCAGCGATGAT<br>CTCTTGGTGGCAGAGGTGGGGATCTCAGACTA<br>TGGTGACAAGCTGAACATGGAGCTGAGTGAGA<br>AGTACAAGCTGGACAAAGAGTCTTACCCAGTC<br>TTCTACCTCTTCCGGGATGGGGACTTTGAGAAT<br>CCTGTCCCATACAGCGGGGCAGTTAAAGTTGG<br>AGCCATCCAGCGCTGGCTCAAGGGGCAGGGAG<br>TCTATCTGGGCATGCCTGGATGTCTGCCTGCGT<br>ACGATGCCCTGGCGGGCCAGTTCATCGAGGCC<br>TCCAGCAGAGAGGCCCGCCAGGCCATCCTGAA<br>ACAGGGGCAGGATGGCCTCTCAGGTGTGAAGG<br>AGACAGACAAGAAGTGGGCCAGTCAGTACCTG<br>AAGATCATGGGGAAGATCTTGGACCAAGGTGA<br>AGACTTCCCGGCCTCCGAGCTGGCCCGGATCA<br>GTAAGCTCATTGAGAACAAGATGAGTGAGGGT<br>AAGAAGGAAGAGCTGCAGAGGAGCCTCAACA<br>TCCTCACCGCCTTCCGCAAGAAAGGCGCCGAG<br>AAGGAGGAGCTCGAAAACCTGTATTTTCAGGG<br>CCACCATCACCATCACCATTA	Forward primer (for amplifying the pETMCSI backbone): GAATTCGAGCTCCCGGGTAC<br><br>Reverse primer (for amplifying the pETMCSI backbone): CATATGTATATCTCCTTCTTAAAGTTAAAC.<br><br>Forward primer (for amplifying the ERp29_TEV_C-His_6 gene): GTTTAACTTTAAGAAGGAGATATACATATGCTGCACACGAAG<br><br>Reverse primer (for amplifying the ERp29_TEV_C-His_6 gene): GTACCCGGGAGCTCGAATTCTTAATGGTGATGGTGATG
MASMTG GB1 _TEV_C-His_6	ATGGCTTCTATGACCGGTATGACCTACAAACTG<br>ATCCTGAACGGTAAAACCCTGAAAGGTGAAAC<br>CACCACCGAAGCGGTTGACGCGGCGACCGCGG<br>AAAAAGTTTTCAAACAGTACGCGAACGACAAC<br>GGTGTTGACGGTGAATGGACCTACGACGACGC<br>GACCAAAACCTTCACCGTTACCGAAGAAAACC<br>TGTATTTTCAGGGCCACCATCACCATCACCAT	Forward primer (for Q32C mutation): GTTTTCAAATGCTACGCGAACGACAACGGTGTTG<br><br>Reverse primer (for Q32C mutation): GCGTAGACTTTGAAAACTTTTTCCGCGGTCGCCG

References

Bergeron, R. J., Wiegand, J., Weimar, W. R., Vinson, J. R. T., Bussenius, J., Yao, G. W., and McManis, J. S.: Desazadesmethyldesferrithiocin analogues as orally effective iron chelators, J. Med. Chem., 42, 95–108, https://doi.org/10.1021/jm980340j, 1998.

Birkofer, L. and Birkofer, A.: β-Aminosäuren, VII. Mitteil.: β-Homocystein, Chem. Ber.-Recueil, 89, 1226–1229, https://doi.org/ 10.1002/cber.19560890522, 1956.

Jeschke, G., Chechik, V., Ionita, P., Godt, A., Zimmermann, H., Banham, J., Timmel, C., Hilger, D., and Jung, H.: DeerAnalysis2006–a comprehensive software package for analyzing pulsed ELDOR data, Appl. Magn. Reson., 30, 473–498, https://doi.org/10.1007/BF03166213, 2006.

Kim, B. C., Kim, K. Y., Lee, H. B., An, J. E., and Lee, K. W.: Production method of intermediate compound for synthesizing medicament, US Patent US8741927B2, 2014.

Stanton-Cook, M. J., Su, X.-C., Otting, G., and Huber, T.: PyParaTools, available at http://comp-bio.anu.edu.au/mscook/PPT/ (last access: 1 May 2022), 2014.

Worswick, S. G., Spencer, J. A., Jeschke, G., and Kuprov, I.: Deep neural network processing of DEER data, Sci. Adv., 4, eaat5218, https://doi.org/10.1126/sciadv.aat5218, 2018.
